# Oxygen Glucose Deprivation Differentially Potentiates Aβ40_E22Q_‐ and Aβ42‐induced Cerebral Endothelial Cell Death, Barrier Dysfunction, and Angiogenesis Impairment

**DOI:** 10.1002/alz70855_103561

**Published:** 2025-12-23

**Authors:** Ashley M Carey, Silvia Fossati

**Affiliations:** ^1^ Lewis Katz School of Medicine, Temple University, Philadelphia, PA, USA; ^2^ Alzheimer's Center at Temple, Lewis Katz School of Medicine, Philadelphia, PA, USA

## Abstract

**Background:**

Disrupted brain hemodynamics and cerebrovascular damage resulting in cerebral hypoperfusion occur early within Alzheimer's Disease (AD) pathogenesis. Cerebral hypoperfusion is also an extremely common consequence of cardiovascular risk factors and diseases (CVRFs/CVDs), which usually manifest in midlife, when AD pathology initiates, and actively contribute to AD progression. Previously our lab has demonstrated that the vasculotropic Dutch mutant, AβQ22, and Aβ42 promote apoptosis, barrier permeability, and angiogenic impairments within cerebral endothelial cells (ECs) and prior research has indicated that hypoperfusion promotes analogous EC dysfunction. Aβ deposition occurs within a hypoperfused environment in AD, but whether exposure of cerebral ECs to Aβ under hypoperfusion conditions results in potentiated increases in cerebral EC dysfunction through activation of common molecular mechanisms remains unknown.

**Method:**

Human cerebral ECs were treated with Aβ40‐Q22 or Aβ42, glucose deprivation (GD), or a combination of both, under normoxia or hypoxia conditions. Cell death mechanisms (apoptosis/necrosis), barrier dysfunction/permeability (TEER/BBB‐regulating proteins/proinflammatory activation), and angiogenesis impairment (vessel branching/VEGF signaling) were evaluated.

**Result:**

Deprivation of glucose and/or oxygen potentiated Aβ‐induced cerebral EC death, barrier instability, junction proteins dysregulation, inflammatory activation, and angiogenesis/wound healing failure. In particular, when in combination with hypoperfusion, AβQ22 exacerbated cerebral EC apoptosis, TEER/ZO1 decreases, ICAM1, IL6, and IL8 upregulation, monocyte migration, and wound healing impairments. Differentially, Aβ42, when in combination with hypoperfusion, more strongly potentiated increases in cerebral EC necrosis and MMP2, phosphorylated claudin‐5, IFNγ, and IL12p70 expression. Additionally, this study identified that GD exerted stronger effects on promoting increases in cerebral EC caspase‐3 activation, apoptosis, and MMP2/ICAM1 expression, while hypoxia demonstrated more robust effects on increasing necrosis, ZO1 expression, and pro‐angiogenic protein expression.

**Conclusion:**

This study reveals specific mechanisms through which hypoxia, low glucose and amyloidosis mutually operate to produce brain EC dysfunction and death, highlighting new potential molecular targets against vascular pathology in comorbid AD/CAA and hypoperfusion conditions.

